# The effect of informal caregiving on medication: evidence from administrative data

**DOI:** 10.1007/s10198-022-01442-0

**Published:** 2022-06-27

**Authors:** Magdalena Stroka-Wetsch

**Affiliations:** Rheinisch-Westfaelisches Institut fuer Wirtschaftsforschung, Essen, Germany

**Keywords:** Informal care, Burden, Drugs, Propensity score matching, I10

## Abstract

This study evaluates the mental and physical strain experienced by informal caregivers. Econometric problems due to individuals selecting themselves into informal care provision are tackled using informative and detailed data on more than 2 million insureds from the largest sickness fund in Germany and applying the propensity score matching technique to estimate the average effect of treatment on the treated. This effect indicates how carers have fared relative to a counterfactual situation in which they would have been non-carers. The radius matching is applied in combination with a strict caliper to obtain a high degree of observational similarity between caring and non-caring individuals. The findings suggest that carers take more psychoactive drugs as well as analgesics and gastrointestinal agents. Females consume about 5 daily defined doses of antidepressants more when they care for dependent relatives. In case of tranquilizers and analgesics, the estimated effect for females amounts about 1 daily defined dose. Considering gastrointestinal agents, the effect amounts to 2 daily defined doses. Thus, informal caregiving appears to be a burdensome task with implications for both mental and physical health.

## Introduction and literature review

Because of rapid developments in medicine and medical technology, chronically, physically and mentally impaired elderly live longer after the onset of illness. The rising life expectancy in general causes an increasing old-age dependency ratio which contributes to rising care prevalence rates [1]. These developments are socially and economically challenging. Apart from the ongoing discussions on the need to improve the quality of formal care and to undertake efforts to avoid dramatic increases of public spending on long-term care services, the role of informal carers is also receiving significant public attention.

The vast majority of long-term care required by impaired people is provided by family and friends. In Germany, 45.6% of all 2.3 million people in need of care were solely attended to by informal caregivers in 2009. Another 23.7% of the dependent persons received out-patient care by formal care services partly combined with care services provided by family and friends [2]. As informal care is the relatively cheaper way of care provision for long-term insurance systems, legal care regulations emphasize the importance of informal caregivers for relieving public budgets [3]. Even though informal caregivers can receive financial compensations for the provision of care services, care laws usually do not consider the opportunity costs resulting from forgone earnings due to decreased working hours or termination of labor supply, as well as decreased productivity, forgone pension entitlements and loss of specific human capital due to the provision of care. Another disregarded cost aspect results from the health effects due to the strain in providing informal care as the carers’ burden can lead to mental and physical morbidity.

While there is strong consensus in the medical and epidemiological literature that the provision of informal care is burdensome and stressful to the caregivers and contributes to mental as well as physical morbidity with potentially detrimental consequences,^1^ there appears to be less evidence in the economic literature. However, the economic perspective is important as a comprehensive discussion about efficiency in long-term care requires knowledge of the full costs of informal caregiving. Therefore, this paper analyzes the question whether there are any costs so far not discussed in the public debate that render informal care provision not as economic as often expected. This could be the case if, e.g., informal care provision goes along with mental or physical health impairments of the informal carers. Other costs include forgone income or human capital for those who leave the labor force to provide informal care. However, the latter costs are not considered in this paper.

The growing economic interest on the health effects of care provision is documented by [13–15], Van den [16, 17], Young Kyung [18, 19] as well as Schmitz and Stroka [20]. While Bobinac et al. [13] provide evidence on a negative effect of informal caregiving on well-being, [15] confirms the negative effect of caregiving on life satisfaction only when using cross-sectional data but does not find any significant effects in a panel data analysis. Coe and van Houtven [14] report negative effects on carers’ mental health and predominantly insignificant results regarding physical health outcomes. This result is confirmed by [18], who analyze the short- and long-term health effects of caregiving and suggest that there are short-term effects on mental health which, however, fade out over time. In contrast, Young Kyung [19] suggests that there is an increased probability of worsened physical health for caregivers caused by the provision of informal care. Van den Berg and Ferrer-i-Carbonell [16] estimate the monetary value of informal care based on the impact that providing care has on individual well-being. According to their calculations, an extra hour of informal care should be compensated by about 10 Euro to maintain the same level of well-being. Van Houtven, Wilson, and Clipp [17] assess the impact of caring on the intake of drugs. One finding is that the intensive care margin is an important factor determining drug intake. This result is confirmed by Schmitz and Stroka [20]. They focus on the double burden resulting from working full-time and the provision of informal care and find evidence for an impaired mental health and a rising health impairment in case of higher care intensity. This paper goes beyond the focus on the working population and considers the carer’s burden in all population groups.

The health effects of informal caregiving is analyzed using the propensity score matching based on administrative data from Germany’s largest statutory sickness fund.^2^ The mental and physical health status is measured in prescribed amounts of certain drugs while differentiating between the care provision for dependent persons and certain levels of care severity. Particularly, this analysis aims at quantifying the effect of caregiving for dependent persons in certain care levels on the amount of prescribed antidepressants, tranquilizers,^3^ analgesics, cardiac and gastrointestinal agents in the course of a year. While the consumption of antidepressants and tranquilizers reflects mental well-being, the other drugs shed light on the physical health status. Hence, the hypothesis that informal care is burdensome is tested focusing on the mental and physical health. Apart from the study by Schmitz and Westphal [18], propensity score techniques were not applied so far in analyses of the health effect of informal care provision.

The findings from the empirical analysis suggest that caregiving has a negative health effect on both the mental and physical health. What is more, the impact on the carers’ mental health increases with the severity of impairment of the cared person measured by care levels.

In the following, the next section describes the data set and reports the relevant descriptive statistics for carers and non-carers. Following the empirical strategy of constructing a balanced sample of carers and comparing individuals without care responsibilities, the third section discusses the estimation of a propensity score equation, before the results of a variety of matching algorithms are presented in the fourth section. The fifth section provides a sensitivity analysis. The paper concludes in the last section. The Appendix documents some data-related issues.

## Data

The empirical analysis is based on data for the periods 2007–2009 provided by the Techniker Krankenkasse (TK), which is the largest statutory sickness fund in Germany with more than 11 million insureds. Like all other sickness funds, the TK collects administrative and claims data on their insureds.^4^ From the large pool of these data, the underlying sample is based on the basic claims data with general socio-demographic information as well as detailed information on prescriptions, diagnoses, care dependency and informal care provision.

The available data include, among others, very detailed information on ascertained diagnoses and prescribed drugs. The latter are measured in daily defined doses (DDD) and are identified using the anatomical therapeutic chemical (ATC) classification. Five different types of drug consumption are considered in this paper, i.e., the yearly sums of prescribed DDDs of antidepressants (ATC: N06A), tranquilizers (ATC: N05B, N05C), analgesics (ATC: N02), cardiac (ATC: C01, C10) and gastrointestinal (ATC: A02–A07, A09) agents.

The information on certain diagnoses is measured dichotomously and equals one if an International Classification of Diseases (ICD-10) code indicating a certain disease is diagnosed at least once either in the out-patient or in-patient health service field in the considered year. The analysis includes diseases of the liver (ICD-10: K70–K77), disorders of thyroid gland (ICD-10: E00–E07), stroke (ICD-10: I61, I63, I64), invasive neoplasms (ICD-10: C00–C97), diseases of the digestive system (ICD-10: K00–K93) and the Parkinson’s disease (ICD-10: G20–G22).

These disease variables control on the one hand for the general health status of the individuals. On the other hand, they also control for diseases that go along with certain drug consumption as diseases of the liver and disorders of thyroid gland can cause depressive symptoms and might lead to prescriptions of antidepressants [21, 22]. Further control variables include the number of hospitalizations, achieved education degree, work position and general socio-demographic outcomes such as gender, age and information on employment.

The variable of primary interest is the carer variable, indicating whether a person provides informal care services to an impaired person or not. This information is available in the data since sickness funds act as both health and long-term care insurance at once and pay legally determined care allowances to informal caregivers. Hence, caregivers have to be reported to the sickness fund to get the allowances that are supposed to compensate their care efforts. This makes it possible to identify informal caregivers in the data and link them to further individual information of these persons as well as on information regarding the care recipients.^5^

This paper does not only concentrate on carers of elderly impaired people but also on people caring for dependent persons of all ages as the burden might occur regardless of the dependent person’s age. While parents usually provide care services to their young dependent children, adult children are likely to care for their frail elder parents. People in the middle and old ages are also likely to be the caregivers of their high-maintenance partners or spouses. As the data also include information on the care level of the dependent person, the variable on the care provision can be broken down into variables indicating care provision for a person in a certain care level. In Germany until 2017, care recipients were classified into three care levels by the Medical Review Board of the Statutory Health Insurance Funds. In 2017, the care levels were modified. In the system until 2017, care level 1 goes along with nursing needs of on average at least 90 min per day, care level 2 includes on average at least 180 min of daily nursing needs. Care level 3 is the most severe care level, indicating average daily care exceeding 300 min. Since the care level of some care recipients is missing in the data, a variable indicating care provision to dependent persons with an unknown care level is also included.

The analysis is based on adult insureds who are at least 35 years old and live in Germany since younger individuals usually do not provide care in Germany and the prescriptions as well as diagnoses information on insureds living abroad might suffer from incompletion.^6^ As a further restriction, observations above the 99th percentile of the dependent variable are excluded to reduce the risk that outliers drive the results.^7^ After applying the mentioned restrictions to the full sample of the available secondary data, the underlying panel data set covers 5,224,552 person-year observations resulting from 2,049,624 individuals observed for a maximum of 3 years.

Table 1 displays some descriptive statistics for carers and non-carers of the pooled sample including all independent variables used in the empirical analysis. Table 2 displays descriptive statistics of the pooled for the dependent variables. Detailed definitions of all variables used are provided in Table 6 in the appendix. Overall, insureds who care for an impaired person take on average more DDDs of all considered drugs compared to insureds without care responsibilities. Carers have also a higher probability of taking any of the considered drug (see Table 2). Notable differences of the means can be observed regarding the pensioner status as well as unemployment and part-time employment. The higher share of part-time employees in the group of carers is not surprising given that care responsibilities are limited by the individuals’ time and energy. In this regard, part-time jobs obviously can be better combined with care tasks. Furthermore, caregivers in general show slightly worse health outcomes concerning certain diseases (see Table 1).

**Table 1:** Descriptive statistics

Variable	Carer		Non-carer	
	Mean	Std. dev	Mean	Std. dev
Dependent variables				
Antidepressants (DDD)	14.926	(73.768)	9.243	(58.959)
Tranquilizers (DDD)	1.823	(16.437)	0.909	(11.768)
Analgesics (DDD)	3.363	(23.516)	2.054	(16.747)
Antihypertensives (DDD)	96.894	(274.968)	91.598	(276.570)
Cardiac agents (DDD)	15.989	(78.390)	15.506	(80.644)
Gastrointestinal agents (DDD)	17.789	(78.390)	14.413	(68.906)
Independent variables				
Information on care provision				
Care provision (all care levels^a^	1.000	(0.000)		
Care provision to person in care level 1^a^	0.240	(0.427)		
Care provision to person in care level 2^a^	0.209	(0.407)		
Care provision to person in care level 3^a^	0.096	(0.295)		
Care provision to person in unknown level^a^	0.454	(0.498)		
Socio-economic characteristics				
Female^a^	0.557	(0.497)	0.400	(0.490)
Age (years)	48.944	(7.858)	47.650	(7.672)
Foreign nationality^a^	0.022	(0.146)	0.027	(0.161)
Employment				
Part-time employed^a^	0.370	(0.483)	0.220	(0.414)
Self-employed^a^	0.002	(0.046)	0.003	(0.059)
Unemployed^a^	0.030	(0.170)	0.022	(0.146)
Temporary unemployed^a^	0.025	(0.155)	0.030	(0.171)
Pensioner^a^	0.032	(0.175)	0.020	(0.142)
Education				
No educational achievement^a^	0.063	(0.242)	0.050	(0.217)
University degree^a^	0.292	(0.455)	0.313	(0.464)
Work position				
Learner^a^	0.001	(0.038)	0.001	(0.032)
Blue-collar worker^a^	0.036	(0.187)	0.047	(0.212)
Craftsman^a^	0.053	(0.224)	0.077	(0.266)
Master craftsman^a^	0.017	(0.129)	0.027	(0.161)
White-collar employee^a^	0.522	(0.500)	0.629	(0.483)
Health status				
Number of hospitalizations	0.127	(0.596)	0.117	(0.476)
Diseases of the liver^a^	0.073	(0.260)	0.065	(0.247)
Disorders of the thyroid gland^a^	0.199	(0.399)	0.156	(0.363)
Stroke^a^	0.006	(0.081)	0.006	(0.075)
Invasive neoplasms^a^	0.057	(0.232)	0.051	(0.220)
Diseases of the digestive system^a^	0.075	(0.263)	0.066	(0.248)
Parkinson’s disease^a^	0.001	(0.034)	0.001	(0.003)
Observations	13,466		5,211,086	

The respective values (multiplied with 100) represent percentage values

^a^Binary variable

**Table 2:** Descriptive statistics—outcome table

Variable	Carer		Non-carer	
	Mean	Std. dev	Mean	Std. dev
Dependent variables in DDD				
Antidepressants	14.9	(73.8)	9.2	(59.0)
Tranquilizers	1.8	(16.4)	0.9	(11.8)
Analgesics	3.4	(23.5)	2.1	(16.7)
Cardiac agents	16.0	(78.4)	15.5	(80.6)
Gastrointestinal agents	17.8	(78.4)	14.4	(68.9)
Dependent variables binary				
Antidepressants^a^	0.1	(0.3)	0.1	(0.2)
Tranquilizers^a^	0.1	(0.2)	0.0	(0.2)
Analgesics^a^	0.1	(0.3)	0.1	(0.3)
Cardiac agents^a^	0.1	(0.2)	0.1	(0.2)
Gastrointestinal agents^a^	0.1	(0.4)	0.1	(0.3)
Person-year observations	13,466		5,211,086	
Individuals	4794		2,044,830	

The respective values (multiplied with 100) represent percentage values

^a^Binary variable

## Empirical strategy

This paper seeks to estimate the average effect of treatment on the treated (ATT), i.e., the average effect on mental and physical health, measured using drug consumption among those who care for an impaired person. The ATT indicates how treated persons (i.e., carers) have fared relative to a counterfactual situation in which these individuals would have not been treated. It is defined as

1 $$E(Y_{{1}} - \, Y_{0} |T \, = {1}),$$

where *T* indicates a binary variable describing the treatment status: specifically *T* = 1 if the subject is an informal caregiver in the considered year, and *T* = 0 otherwise.

Since care provision histories are not subject to random assignment, the analysis rests primarily on the conditional independence assumption (CIA) also referred to as “selection-on-observables” [23]. This assumption states that

2 $$Y_{0} \bot T\left| W \right.,$$

where *W* is a set of observable variables. It corresponds to the assumption that after conditioning on a set of observable covariates, potential health outcomes would be the same for those who care and those who do not care for impaired persons. The extensive information on personal characteristics included in the underlying data set covers the necessary range of observables to render this empirical strategy viable.

The overlap or common support assumption is given by

3 $${\text{Pr}}(T = {1}|W) < {1}.$$

This assumption requires that, for each treated unit, there are control units with the same *W*. Hence, under the CIA and overlap condition, the ATT can be identified as

4 $$E(Y_{{1}} - Y_{0} |T = { 1}) \, = E\left( {E\left( {Y_{{1}} - Y_{0} |T = { 1},W} \right)} \right) \, = E(E(Y_{{1}} |T = { 1},{\text{ W}}) \, {-}E(Y_{0} |T = \, 0,W)|T = { 1}).$$

To identify the ATT, a relatively small number of observations of carers (13,466 person-year observations) are compared to a much larger number of observations of non-carers (5,211,086 person-year observations) by applying the propensity score method. This method extracts a control group from the whole sample of non-carers in which the distribution of covariates is similar to the distribution in the treatment group. This selection of the group of controls is done through a two-step procedure. In the first stage, a logit model is used to estimate the conditional probability of being a caregiver given a vector of observed covariates which may affect the probability of being a caregiver. The estimated conditional probability is the propensity score, which is used in the second step, where each carer is matched to a non-carer that has the closest propensity score. This matching procedure can be performed using different matching algorithms, i.e., the kernel, radius and nearest-neighbor matching with and without replacement, with an emphasis on their ability to ascertain the desired balancing.

While in small samples, the choice of the matching approach may be important [24], with growing sample sizes, all matching approaches become closer to exact matching and should yield asymptotically the same result [25]. Hence, only the radius matching with a caliper of width 0.00001 is presented in this paper. With the fairly strict caliper, it is possible to require a high degree of observational similarity between treatment and control cases and still find matching control cases for the treatment cases. Nevertheless, all other variations on these themes (regarding the caliper) generate very similar results.^8^ Since the literature suggests gender differences in the provision of informal care (see e.g., [26] separate regressions are carried out for women and men. The underlying samples used in the matching approach are trimmed to those observations that lie on the common support.

To shed light on the question whether the mental and physical impact on the caregivers increases with the severity of impairment of the cared person, the ATT for carers in certain care levels are estimated in separate models (i.e., including only individuals caring for persons in either care level 1, 2 or 3 in the treatment and non-carers in the control groups). The estimation procedure is the same as in the basic model described above taking observable covariate differences into account. However, since only carers of dependent persons in a certain care level are considered and compared with non-carers, the sample sizes vary due to the exclusion of the carers of dependent persons in other care levels.

## Results

### Matching quality

As the propensity score matching can only lead to credible estimates of the effects of treatment if the desired balancing of observable covariates is achieved, standard *t*-tests for equality of means in the treatment and comparison groups, after matching on the scores, were performed for every specification. Table 3 demonstrates that this approach is very successful in this regard, leading to a complete covariate balance.^9^

**Table 3:** Covariate balance—individual *t*-test

Variable	Males		Females	
	Test values for unmatched	Test values for matched	Test values for unmatched	Test values for matched
Socio-economic characteristics				
Age^a^	17.7***	− 0.1	16.3***	− 0.3
Foreign nationality^a^	− 1.4	0.2	− 3.6***	0.2
Employment				
Part-time employed	15.7***	1.1	21.8***	0.1
Self-employed	− 1.3	0.2	− 1.9*	0.3
Unemployed^a^	3.0***	− 0.2	4.7***	0.5
Temporary unemployed^a^	1.4	− 0.1	3.4***	− 0.2
Pensioner	10.5***	1.3	4.8***	0.9
Education				
No educational achievement^a^	2.7***	0.1	4.5***	0.1
University degree	3.5***	− 0.4	− 3.5***	− 0.6
Work position				
Learner	1.5	0.1	0.0	0.1
Blue-collar worker	− 2.5***	− 0.4	− 2.6***	− 0.2
Craftsman	− 4.1***	0.5	− 2.5***	0.8
Master craftsman	− 3.1***	− 0.4	0.2	− 0.1
White-collar employee	− 3.6***	− 0.8	− 20.0***	− 0.2
Health status				
Number of hospitalizations^a^	0.3	0.1	2.4***	0.4
Diseases of the liver^a^	3.6***	0.2	5.6***	0.4
Disorders of the thyroid gland	3.9***	− 0.1	3.1***	1.6
Stroke	2.7***	0.2	0.2	0.0
Invasive neoplasms	0.9	0.0	1.8*	0.3
Diseases of the digestive system	4.0***	0.2	5.6***	0.6
Parkinson’s disease	1.2	0.1	1.6	0.2
Year^a^	− 11.4***	1.1	− 13.5***	0.3

Significant at ***: 1% level; **: 5% level; *: 10% level

^a^Significant variable in the logistic regression

A final check of the quality of the matching procedure comes from a comparison of the distribution of the propensity scores of the carers and non-carers. The results show that there are no common support problems. There are many controls for each carer within small intervals of the estimated propensity scores. Overall, the distribution of the matched non-carers resembles the distribution of carers, and there is thus an overlap in the estimated propensity scores. Hence, it can be concluded that the propensity score approach works very well.^10^

### Matching results

The estimate of the effect of care provision on mental and physical health is based on a comparison of the drug intake of carers and non-carers. Table 4 provides evidence from the radius propensity score matching on the differences in drug intake between these two groups. While the first column of Table 4 reports the estimates for males, the last column reports the results for females. Overall, the matched comparisons tend to confirm the unmatched comparisons quite closely. The intakes of antidepressants, tranquilizers, analgesics and gastrointestinal agents are higher for carers than for non-carers. The differences are substantial and amount, in the case of antidepressants, up to about 5 DDD per year for men. Hence, care provision goes along with an antidepressant intake increase by almost 60% (see Table 2), such that the economic significance of the results is high. As a robustness check different matching algorithms (kernel as well as nearest-neighbor with and without replacement) were applied and are available upon request. While the point estimates of the effect of caregiving on antidepressants intake by females tend to be slightly smaller in absolute values when employing other matching techniques than the nearest-neighbor with replacement, overall the results are robust, both quantitatively and qualitatively. This confirms that the requirements of the matching regarding a large sample size are fulfilled and the results tend to exact matching.

**Table 4:** Treatment effect of care provision

	Effect for males	Effect for females
Antidepressants	4.9***	3.7***
	(0.9)	(0.9)
Tranquilizers	0.7***	0.8***
	(0.2)	(0.2)
Analgesics	1.2***	0.8***
	(0.3)	(0.3)
Cardiac agents	− 1.1	0.5
	(1.3)	(0.7)
Gastrointestinal agents	3.0***	2.0**
	(1.1)	(0.8)
Untreated on support	3,125,140	2,085,946
Treated on support	5,696	7,495

Significant at ***: 1% level; **: 5% level; *: 10% level; standard errors in parentheses. In case of males and females, one treated off support

In the next step, the effects of caring for dependent persons in certain care levels compared to non-carers are considered. The results reported in Table 5 are limited to antidepressants and tranquilizers. The other specifications mostly do not reveal significant differences across care intensities. The effects for antidepressants and tranquilizers increase and gain significance the higher the care level is. Considering antidepressants, in the most severe care level, the effect is twice higher compared to the lowest level. Accordingly, carers of impaired persons in care level 3 consume on average about 8–10 DDDs more of antidepressants per year than non-carers.

**Table 5:** Treatment effect of care provision by care level

	Effect for males	Effect for females
Antidepressants		
Care level I	4.4**	4.5***
	(2.0)	(1.9)
Care level II	7.5***	5.9***
	(2.2)	(2.2)
Care level III	10.4***	8.3***
	(3.7)	(3.0)
Unknown care level	3.2***	1.1
	(1.2)	(1.1)
Tranquilizers		
Care level I	0.1	1.0**
	(0.3)	(0.5)
Care level II	0.6	0.6
	(0.6)	(0.5)
Care level III	1.2	2.7***
	(0.7)	(0.9)
Unknown care level	0.8***	0.3
	(0.3)	(0.2)

Significant at ***: 1% level; **: 5% level; *: 10% level: standard errors in parentheses. Treated males in care level 1: 145, untreated: 984,439; treated males in care level 2: 69, untreated: 955,328; treated males in care level 3: 110, untreated: 958,760; treated males in unknown care level: 180, untreated: 984,762; treated females in care level 1: 204, untreated: 641,477; treated females in care level 2: 163, untreated: 652,989; treated males in care level 3: 92, untreated: 657,368; treated females in unknown care level: 57, untreated 644,936. All treated and untreated on support

To get an idea of the results in terms of costs from the payer’s perspective (the insurance companies), a back-on-the-envelope calculation is performed focusing on antidepressants and extrapolating the results to the entire German population. In a subsample^11^ of TK insureds in 2009, the average price for one DDD of antidepressants amounts to 0.81€ (the standard deviation is 0.77 and the price range goes from 0.17€ to 6.35€). As it is well documented in the literature that most caregivers are female (see e.g., [27]), this paper concentrates on the results for females and consider the results from the propensity score matching without the differentiation of the care levels.^12^ Taking the lowest obtained result for women (3.73)^13^ and multiplying it by all informal caregivers in Germany in 2009 (1,620,762), the costs resulting from the higher antidepressant intake are almost 5 million €.^14^ Note that only the number of dependent persons who are solely cared by their family and friends is considered in this calculation. However, another 555,198 dependent person received out-patient care by their family and friends and/or formal care services.

## Sensitivity analysis

As mentioned above, the estimation of treatment effects with matching estimators is based on the CIA. Thus, if the treated and non-treated differ in unobserved characteristics, the results reported above may be biased. This potential problem is addressed with the bounding approach proposed by Rosenbaum [28]. This approach calculates upper and lower bounds on the test statistics used to test the hypothesis of no care effect for different values of hidden bias, i.e., it determines how strongly an unobserved variable must influence the selection process to undermine the implications of the matching analysis. By comparing the Rosenbaum bounds on treatment effects, it is possible to assess the strength that the unmeasured heterogeneity or endogeneity would require so that the obtained effects from the matching analysis would have arisen solely through selection effects. In the underlying case, the test shows that the robustness to hidden bias varies considerably across the outcome variables. While the results regarding antidepressants and tranquillizers are very robust to hidden bias, this is not the case considering analgesics and gastrointestinal agents (detailed results are available upon request).

## Discussion

This paper uses the propensity score matching approach to empirically compare the drug intake of carers and non-carers, taking observable covariate differences into account. Considering the precisely measured amounts of prescribed drugs instead of diagnoses allows me to account for the severity of certain diagnoses. This goes along with the advantage of more detailed information on the magnitude of health impairments.

Identification of the health effects of care provision, however, comes with particular challenges. The first challenge is the data, which should provide individual information on reliable health outcomes, care responsibilities as well as socio-demographic characteristics. It is argued above and below that the administrative data from the TK, with very detailed information on more than 5.2 million person-year observations can be used for such an analysis. The second challenge comes from individual self-selection into informal care provision. For example, if individuals in good health conditions choose to provide care for their family and friends, then a comparison of the health outcomes of individuals with and without care responsibilities will not be informative about the casual care effect. The fact that selection into caregiving is not random causes selection bias, which is intended to be solved by conditioning on observable variables that represent the confounding factors, i.e., propensity score matching techniques are employed to make carers and non-carers comparable.

There are some important aspects of the underlying data and method. First, the analysis is based on claims data generated by experts of health such as physicians. This goes along with obvious advantages of this data set resulting from its large sample size. Hence, the estimates are more precise. However, as the data were documented for billing purposes, possible mechanisms like up-coding might play a role in the data and reduce its objectivity. However, if this is the case, one might expect rather a general influence of the billing purpose on the data without selective impacts. Finally, this study builds on prior research using more recent data. However, one issue in the data is that individuals are identified as caregivers only if they provide care to a family member who is also insured by the TK. Nevertheless, it is possible that children from adult impaired persons are insured by other insurance companies. In fact, only 0.25% of the underlying sample members can be identified as caregivers while one would expect a higher number, since about 1.25% of the German population receive informal care by family members [2]. Hence, a considerable amount of individuals is potentially assigned into the control group “no care provision” although they provide care. This is considered to be a minor problem as the relative number of caregivers that is mistakenly put into the control group is negligible compared to those who indeed do not provide care. Thus, the mistakenly included caregivers in the control group should not affect the overall mean effects in this group. If it does affect the results and if care provision goes along with worse health, this would lead to an underestimation of the true relation (in absolute values). The total number of 7634 identifiable caregivers is still much larger than in studies that rely on survey data.

Although the administrative data set has certain advantages, it did not allow to address some limitations. One problematic issue in the analysis is that the caregiver’s health may be affected by both the provision of care and the fact that a loved person is impaired. In the latter case, health outcomes could be affected by the mental strain even if no care service is provided by oneself to the impaired person. If persons with dependent relatives but without care responsibilities suffer from the mental strain that results in higher drug intake, the effect in this paper—that is considered as the total effects resulting from the physical afford of caring and the mental strain of facing the fact that a loved person is impaired—could be underestimated because it is not possible to observe these persons in the data. Moreover, the data do not include any economic information (expect work position) and due to sharpened data protection regulations, it is not possible to get the data for more up-to-date periods.^15^ Considering the outcome variables, it is important to mention that prescribed drugs are not necessary consumed drugs. However, only those prescriptions are included in the data that were fetched in pharmacies, nevertheless, even though it remains unclear whether these drugs were consumed.

The empirical analysis in the case of Germany, a country that is the largest in Europe with a pronounced social security system, and subject to a strong demographic change, reveals that both female and male carers seem to have a higher drug intake of antidepressants, tranquilizers, analgesics and gastrointestinal agents. The effects on the mental health even increases with the care severity of the dependent individuals. However, this is not the case regarding physical health. In the case of cardiac agents, no significant results could be found at all. These results are in line with [14], who find evidence for immediate negative effects on carers’ mental health and negative effects regarding physical health only in the long run. As shown by [20], the mental health effect can also be explained by the double burden of caring and working full-time in case of the working population.

As the propensity score matching is based on observables, unobserved confounders might remain a problem. People are not randomly assigned to informal care and emotional aspects, the income, etc. might be confounders in this context. Even though the propensity score matching is based on a long list of observables due to the possible confounders, the results might be rather considered as associations.

The German government recently acknowledged that a realignment of the care system is necessary. This analysis contributes to the current debate on how to realign the care system in Germany and countries with similar demographic developments. Certainly, this paper does not postulate that informal home care should be fully replaced by professional care due to increased drug intake of caregivers. This paper does not provide a full cost–benefit analysis of different types of care to decide which one is the best from an economic perspective. However, one should note that the results suggest that the costs for informal family care are higher than usually assumed.

## Appendix

See Table 6.

**Table 6:** Definition of variables

Variable	Description
Dependent variables	
Antidepressants	Sum of prescribed DDDs of antidepressants in the considered year
Tranquilizers	Sum of prescribed DDDs of tranquilizers. sedatives and hypnotics in the considered year
Analgesics	Sum of prescribed DDDs of analgesics in the considered year
Antihypertensive agents	Sum of prescribed DDDs of antihypertensive agents in the considered year
Cardiac agents	Sum of prescribed DDDs of cardiac agents s in the considered year
Gastrointestinal agents	Sum of prescribed DDDs of gastrointestinal agents in the considered year
Independent variables	
Information on care provision	
Care provision (all care levels)	= 1 if care provision to impaired person in any care level. 0 otherwise
Care provision to person in care level 1	= 1 if care provision to impaired person in care level 1. 0 otherwise
Care provision to person in care level 2	= 1 if care provision to impaired person in care level 2. 0 otherwise
Care provision to person in care level 3	= 1 if care provision to impaired person in care level 3. 0 otherwise
Care provision to person in unknown level	= 1 if care provision to impaired person in unknown care level. 0 otherwise
Socio-demographic characteristics	
Female	= 1 if female. 0 otherwise
Age	age of individual
Foreign nationality	= 1 if not German. 0 otherwise
Self-employed	= 1 if self-employed. 0 otherwise (reference group: employed)
Part-time worker	= 1 if part-time worker or home worker. 0 otherwise
Unemployed	= 1 if unemployed. 0 otherwise (reference group: employed)
Temporary unemployed	= 1 if unemployed up to 150 days in a year. 0 otherwise
Pensioner	= 1 if pensioner. 0 otherwise (reference group: employed)
Education	
No educational achievement	= 1 if no educational achievement. 0 otherwise (reference group: professional education)
University degree	= 1 if university degree. 0 otherwise (reference group: professional education)
Work position	
Apprentice	= 1 if learner. 0 otherwise
Blue-collar worker	= 1 if blue-collar worker. 0 otherwise
Craftsman	= 1 if craftsman. 0 otherwise
Master craftsman	= 1 if master craftsman. 0 otherwise
White-collar employee	= 1 if white-collar employee. 0 otherwise
Health status	
Number of hospitalizations	Number of hospitalizations in the considered year
Diseases of the liver	= 1 if diseases of the liver were diagnosed in the considered year. 0 otherwise
Disorders of the thyroid gland	= 1 if disorders of the thyroid gland were diagnosed in the considered year. 0 otherwise
Stroke	= 1 if stroke was diagnosed in the considered year. 0 otherwise
Invasive neoplasms	= 1 if invasive neoplasms were diagnosed in the considered year. 0 otherwise
Diseases of the digestive system	= 1 if diseases of the digestive system were diagnosed in the considered year. 0 otherwise
Parkinson’s disease	= 1 if stroke was diagnosed in the considered year. 0 otherwise

## Footnotes

1 Examples for evidence of the mental burden are given, e.g., in [4–6]. Especially depression and other symptoms of psychological distress are considered as major consequences of care giving [7–9]. Furthermore, recent studies indicate that informal care provision is associated with poor physical health outcomes in addition to the adverse mental health [10]. It has been shown that caregivers are at increased risk for developing hypertension [11] and cardiovascular diseases [12]. However, the empirical strategy of the existing studies is limited to descriptive comparisons and multivariate regressions.

2 The size and detailed degree of information of the administrative data allows to answer the underlying question more precisely than other available data sources.

3 Specifically, this group is “tranquilizers, sedatives and hypnotics”, but in the following, it is referred to as tranquilizers for brevity.

4 The original data are structured by payment sections as the data are collected for billing reasons. Using an individual identifier for each insured, a full sample of the data was prepared for the underlying analysis by the author of the study at the TK in Hamburg.

5 In both data sets (claims and care data), the insureds have the same id, which allows me to merge the data.

6 Due to the fact that the outcome variables are measured on a yearly basis, observations on individuals not observed throughout a complete year are excluded to avoid any measurement bias.

7 However, the results are robust to not trimming the data.

8 In particular, smaller values (from 0.001 onwards) of caliper, respectively, were considered.

9 The results from the t-tests for equality of means in the treatment and comparison groups after matching for certain care levels are not presented in the interest of brevity. However, in these subsamples, complete covariate balances were achieved as well.

10 Figures on the density distribution are available upon request.

11 The subsample includes only insureds consuming antidepressants in 2009.

12 In the calculation, it is assumed that all caregivers are females. However, as the results for men and women differ only slightly this assumption should not influence the calculated result significantly.

13 As the estimated effects for men are only slightly lower, the calculated amount of costs should not change significantly when taking male carers into account.

14 Calculation: 3.73 DDD* 0.81€ per DDD*1,620,762 informal carers = 4,896,808€.

15 The patient claims were pseudonymized by the TK and no key for de-identification was provided. The study was approved by the data protection office and the ethics committee.
